# Antimicrobial Resistance Genes and Diversity of Clones among Faecal ESBL-Producing *Escherichia coli* Isolated from Healthy and Sick Dogs Living in Portugal

**DOI:** 10.3390/antibiotics10081013

**Published:** 2021-08-20

**Authors:** Isabel Carvalho, Rita Cunha, Carla Martins, Sandra Martínez-Álvarez, Nadia Safia Chenouf, Paulo Pimenta, Ana Raquel Pereira, Sónia Ramos, Madjid Sadi, Ângela Martins, Jorge Façanha, Fazle Rabbi, Rosa Capita, Carlos Alonso-Calleja, Maria de Lurdes Nunes Enes Dapkevicius, Gilberto Igrejas, Carmen Torres, Patrícia Poeta

**Affiliations:** 1 Microbiology and Antibiotic Resistance Team (MicroART), Department of Veterinary Sciences, University of Trás-os-Montes and Alto Douro, 5000-801 Vila Real, Portugal; isabelbarrosocarvalho@utad.pt; 2 Department of Genetics and Biotechnology, University of Trás-os-Montes and Alto Douro, 5000-801 Vila Real, Portugal; gigrejas@utad.pt; 3 Functional Genomics and Proteomics Unit, University of Trás-os-Montes and Alto Douro, 5000-801 Vila Real, Portugal; 4 Laboratory Associated for Green Chemistry (LAQV-REQUIMTE), New University of Lisbon, 2829-516 Monte da Caparica, Portugal; 5 Area Biochemistry and Molecular Biology, University of La Rioja, 26006 Logroño, Spain; sandra.martinezal@alum.unirioja.es (S.M.-Á.); chenoufns@gmail.com (N.S.C.); sadimadjid@gmail.com (M.S.); carmen.torres@unirioja.es (C.T.); 6 Hospital Veterinário Cascais da Onevet, 2775-352 Parede, Lisbon, Portugal; ritalaurentinocunha@gmail.com; 7 Clínica Veterinária do Vouga, 3740-253 Sever do Vouga, Portugal; clinica.vouga@gmail.com; 8 Laboratory of Exploration and Valuation of the Steppe Ecosystem, University of Djelfa, Djelfa 17000, Algeria; 9 Hospital Veterinário de Trás-os-Montes, 5000-056 Vila Real, Portugal; paulo.pimenta@onevetgroup.pt; 10 Centro Veterinário de Macedo de Cavaleiros, 5340-202 Bragança, Portugal; vetcenterbrg@gmail.com; 11 VetRedondo, Consultório Veterinário de Monte Redondo Unipessoal Lda, Monte Redondo, 2425-618 Leiria, Portugal; soniacatarinaramos@gmail.com; 12 Laboratory of Biotechnology Related to Animals Reproduction, Université Saad Dahlab de Blida, Blida 09000, Algeria; 13 Animal and Veterinary Research Center (CECAV), University of Trás-os-Montes and Alto Douro, 5000-801 Vila Real, Portugal; angela@utad.pt; 14 Centro Veterinário Jorge Façanha, 5140-060 Carrazeda de Ansiães, Portugal; jorgefacanha@hotmail.com; 15 Australian Computer Society, Docklands, Melbourne, VIC 3008, Australia; bilirabbi@yahoo.com; 16 Department of Food Hygiene and Technology, Veterinary Faculty, University of León, 24071 León, Spain; rosa.capita@unileon.es (R.C.); carlos.alonso.calleja@unileon.es (C.A.-C.); 17 Institute of Food Science and Technology, University of León, 24071 León, Spain; 18 Faculty of Agricultural and Environmental Sciences, University of the Azores, 9500-321 Angra do Heroísmo, Portugal; maria.ln.dapkevicius@uac.pt; 19 Institute of Agricultural and Environmental Research and Technology (IITAA), University of the Azores, 9500-321 Angra do Heroísmo, Portugal

**Keywords:** antimicrobial resistance, dogs, *Escherichia coli*, ESBL, CTX-M-15, CTX-M-1, CTX-M-32, CTX-M-55, CTX-M-14, qAmpC, CMY-2

## Abstract

The purpose of this study was to analyse the prevalence and genetic characteristics of ESBL and acquired-AmpC (qAmpC)-producing *Escherichia coli* isolates from healthy and sick dogs in Portugal. Three hundred and sixty-one faecal samples from sick and healthy dogs were seeded on MacConkey agar supplemented with cefotaxime (2 µg/mL) for cefotaxime-resistant (CTX^R^) *E. coli* recovery. Antimicrobial susceptibility testing for 15 antibiotics was performed and the ESBL-phenotype of the *E. coli* isolates was screened. Detection of antimicrobial resistance and virulence genes, and molecular typing of the isolates (phylogroups, multilocus-sequence-typing, and specific-ST131) were performed by PCR (and sequencing when required). CTX^R^ *E. coli* isolates were obtained in 51/361 faecal samples analysed (14.1%), originating from 36/234 sick dogs and 15/127 healthy dogs. Forty-seven ESBL-producing *E. coli* isolates were recovered from 32 sick (13.7%) and 15 healthy animals (11.8%). Different variants of *bla*_CTX-M_ genes were detected among 45/47 ESBL-producers: *bla*_CTX-M-15_ (*n* = 26), *bla*_CTX-M-1_ (*n* = 10), *bla*_CTX-M-32_ (*n* = 3), *bla*_CTX-M-55_ (*n* = 3), *bla*_CTX-M-14_ (*n* = 2), and *bla*_CTX-M_-variant (*n* = 1); one ESBL-positive isolate co-produced CTX-M-15 and CMY-2 enzymes. Moreover, two additional CTX^R^ ESBL-negative *E. coli* isolates were CMY-2-producers (qAmpC). Ten different sequence types were identified (ST/phylogenetic-group/β-lactamase): ST131/B2/CTX-M-15, ST617/A/CTX-M-55, ST3078/B1/CTX-M-32, ST542/A/CTX-M-14, ST57/D/CTX-M-1, ST12/B2/CTX-M-15, ST6448/B1/CTX-M-15 + CMY-2, ST5766/A/CTX-M-32, ST115/D/CMY-2 and a new-ST/D/CMY-2. Five variants of CTX-M enzymes (CTX-M-15 and CTX-M-1 predominant) and eight different clonal complexes were detected from canine ESBL-producing *E. coli* isolates. Although at a lower rate, CMY-2 β-lactamase was also found. Dogs remain frequent carriers of ESBL and/or qAmpC-producing *E. coli* with a potential zoonotic role.

## 1. Introduction

Antimicrobial resistance has become a major challenge for public health worldwide. The selective pressure, which results from the long-term use of antibiotics, allowed bacterial species to be resistant to these agents. It has been believed that this resistance is reaching alarming levels, considering that resistance rates have risen extremely, during the last two decades [1,2].

*Escherichia coli*, a Gram-negative bacterium belonging to the *Enterobacteriaceae* family, is a common member of the intestinal microbiota of humans and companion animals [3,4]. However, this opportunistic pathogen can cause intestinal and extra-intestinal diseases. It may contribute, in many cases, to antimicrobial resistance dissemination. Recently, the World Health Organization [5] published a global priority list of antibiotic-resistant bacteria, where third-generation cephalosporin- and/or carbapenem-resistant *Enterobacteriaceae*, including *E. coli*, were included in the Priority 1 group. It is important to note that first-generation cephalosporins and amoxicillin + clavulanic acid are among the most prescribed drugs for dogs [3,4,6].

During recent years, the emergence and rapid dissemination of *Enterobacteriaceae* carrying genes encoding the extended-spectrum-β-lactamases (ESBLs), acquired AmpC β-lactamases (qAmpC), or carbapenemases are considered of great concern [4,7]. One of the most important mechanisms is the plasmid-mediated production of extended-spectrum β-lactamases (ESBLs), which can hydrolyse broad-spectrum cephalosporins (such as cefotaxime). The horizontal gene transfer (HGT) among bacteria is driven by plasmids [8,9], which play an important role in the transference of antibiotic-resistance genes among bacteria, contributing to the spread of multidrug resistance (MDR), and limiting therapeutic options [10]. ESBLs of the CTX-M-type and the qAmpC CMY-2 are increasingly being reported in bacteria worldwide, while livestock or companion animals are potential sources, leading to the spread of β-lactam-resistant bacteria in humans [11,12]. 

The close proximity between dogs and their owners increases the possibility of transmitting resistant bacteria [13,14]. According to Dupouy et al. [6], dogs could transmit MDR bacteria due to their close contact with humans, the high consumption of β-lactams in small animal veterinary practice, and also the frequent occurrence of ESBL/qAmpC-producing *E. coli*. The occurrence of ESBL-producing *E. coli* has been widely reported in both healthy companion animals [12,15] and diseased ones [1,16,17,18,19]. International high-risk clones of *E. coli* are frequently detected worldwide, not only in human infections but also in those of companion animals [2,3,17]. Over the past 5 years, the presence of ESBL/qAmpC genes in *Enterobacteriaceae* strains from faeces of dogs in Europe has been reported in several studies [6,12,13,20], including Portugal [21,22]. However, knowledge about the clonality of ESBL/qAmpC-producing isolates and the potential zoonotic reservoir of human-associated STs is not well documented. Moreover, there is still a lack of data about their prevalence in sick and healthy dogs, simultaneously. In this study, we aim at characterizing the prevalence and diversity of ESBL- and qAmpC- producing *E. coli* faecal isolates from healthy and sick dogs in Portugal, as well as determining their genetic lineages and phylogenetic groups. 

## 2. Materials and Methods

### 2.1. Animals and Sampling

A total of 361 faecal samples were recovered from 127 healthy and 234 hospitalized dogs from different cities in Portugal. All samples were collected between April andAugust 2017 (one sample/animal) using standardized procedures [23]. 

The hospitalized dogs came from 7 different veterinary hospitals or clinic centers; the healthy dogs came from a local kennel located in Vila Real (*n* = 31) and from local houses (*n* = 96). The seven hospitals/clinic centers were located in different centers of the Portuguese territory: Bragança (1 hospital, *n* = 29 dogs), Vila Real (4 hospitals, *n* = 62), Aveiro (1 hospital, *n* = 58), Leiria (1 hospital, *n* = 17), and Lisbon (1 hospital, *n* = 68) (Figure S1). It is important to note that faecal samples from unhealthy dogs were collected from the ordinary population of animals hospitalized in hospitals or veterinary clinics, not endangering their health, or causing harm or pain. In the same line, faecal samples from healthy animals were also recovered by their owners. All of them were analysed with the owner’s permission or with kennel collaboration. The faecal samples were dispatched immediately to the Microbiology Laboratory of the University of Trás-os-Montes and Alto-Douro (UTAD).

### 2.2. E. coli Isolation

From each faecal sample, a small portion of 2 g was diluted in Brain Heart Infusion (BHI, Condalab, Spain) and incubated in aerobic conditions for 24 h at 37 °C. After that, samples were seeded on MacConkey agar (Becton, Dickinson and Company Sparks, Le Pont de Claix, France) supplemented with cefotaxime (2 µg/mL) and incubated for 24 h at 37 °C. Colonies showing *E. coli* morphology were recovered (one colony per sample) and identified by a classical biochemical method named IMViC (Indol, Methyl-red, Voges–Proskauer, and Citrate).

The matrix-assisted laser desorption/ionization time-of-flight mass spectrometry method (MALDI-TOF MS, MALDI Biotyper® from Bruker Daltonik, Bremen, Germany) was applied in this study to confirm bacterial species identification. *E. coli* isolates were kept at −80 °C and were further characterized.

### 2.3. Susceptibility Testing

Antimicrobial susceptibility testing was performed using the Kirby–Bauer disk diffusion method and according to Clinical and Laboratory Standards Institute guidelines (2019) [24] for the following 15 antibiotics (μg/disk): ampicillin (10), amoxicillin + clavulanic acid (20), cefotaxime (30), cefoxitin (30), ceftazidime (30), aztreonam (30), imipenem (10), gentamicin (10), streptomycin (10), ciprofloxacin (5), trimethoprim-sulfamethoxazole (1.25 ± 23.75), amikacin (30), tobramycin (10), tetracycline (30), and chloramphenicol (30). In addition, the screening of phenotypic ESBL production was carried out by the double-disk synergy test using cefotaxime, ceftazidime, and amoxicillin/clavulanic discs in Mueller Hinton (MH) agar (Condalab, Spain) [24]. 

### 2.4. DNA Extraction and Quantification

Genomic DNA from cefotaxime-resistant (CTX^R^) isolates were extracted using the boiled method [25]. In order to quantify the nucleic acid concentration and the level of purity, the absorbance readings were taken at 260 and 280 nm (Spectrophotometer ND-100, Nanodrop, Thermo Fisher Scientific, Waltham, MA USA).

### 2.5. Antibiotic Resistance and Virulence Genes Detection

The genetic basis of resistance was investigated using PCR methods and subsequent sequencing of the obtained amplicons (specific genes). Negative and positive controls of the University of La Rioja were used in this work. Moreover, the data regarding PCR conditions for each primer (Sigma-Aldrich, Madrid, Spain) as well as the size of the obtained amplicons that were sequenced are illustrated in detail in Table S1. 

The presence of *bla*_CTX-M_ (Groups 1 and 9), *bla*_CMY-2_, *bla*_DHA-1_, *bla*_TEM_, *bla*_SHV_, *bla*_VEB_*, bla*_KPC2/3_, *bla*_NDM,_ *bla*_OXA-48,_ and *bla*_VIM_ was tested by PCR/sequencing (Table S1) [26,27,28,29,30]. Furthermore, the *mcr*-1 gene (colistin resistance) [31], *tet*A/*tet*B (tetracycline resistance) [32], *stx*_1,2_ genes related to Shiga toxin-producing *E. coli* (STEC) [33], and *int*1 gene (integrase of class 1 integrons) and its variable region (RV *int*1) were also analysed by PCR/sequencing [30]. Analysis of DNA sequences was performed using the standard databases (nucleotide collection) in the BLASTN program (2021 version), available at the National Center for Biotechnology Information (https://blast.ncbi.nlm.nih.gov/Blast.cgi (accessed on 31 January 2021). 

### 2.6. Multilocus Sequence Typing and Phylogroup Typing of E. coli Isolates

Multilocus sequence typing (MLST), by the analysis of seven housekeeping genes (*fum*C, *adk, pur*A, *icd, rec*A, *mdh*, and *gyr*B), was carried out for thirteen representative *E. coli* isolates (based on the antimicrobial resistance phenotype) according to the protocol described on PubMLST (Public databases for molecular typing and microbial genome diversity) website [34]. The allele combination was determined after sequencing of the seven genes, and the sequence type (ST) and clonal complex (CC) were identified.

Phylogenetic classification of all *E. coli* isolates was performed according to the presence of *chu*A, *yja*A, and TSPE4.C2 genes [35].

### 2.7. Statistical Analyses

All statistical analyses were performed using the JMP Statistics software (v7.0, SAS Institute). The Pearson’s Chi-square and Fisher’s exact tests were performed to understand and identify the associations between the origin of strain (healthy or sick dog) and antibiotic resistance (antibiotic and gene). In this line, we consider two categorical variables: the sick or healthy animal, and the resistance for each antibiotic/gene. A *p*-value < 0.05 was established as indicating statistical significance [36].

## 3. Results

CTX^R^ *E. coli* isolates were recovered in 51/361 faecal samples tested (14.1%), originating from 36/234 sick dogs (15.4%) and 15/127 healthy dogs (11.8%). These CTX^R^ isolates were detected among 29 male dogs (56.9%) and 22 female dogs (43.1%); most of them belonged to an undetermined breed (*n* = 38), followed by the Labrador/Golden Retriever breed (*n* = 4), while the remaining dogs belonged to different pure breeds (Table 1 and Table 2). 

**Figure 1:**
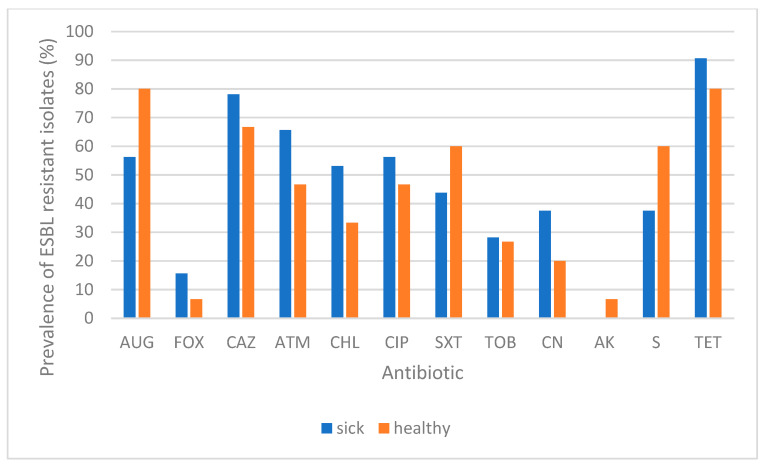
Prevalence of antibiotic-resistance among ESBL-producing *E. coli* isolates in sick and healthy dogs. Antibiotics tested: AUG, amoxicillin-clavulanic acid; FOX, cefoxitin; CAZ, ceftazidime; ATM, aztreonam; CHL, chloramphenicol; CIP, ciprofloxacin; SXT, trimethoprim-sulfamethoxazole; TOB, tobramycin; CN, gentamicin; AK, amikacin; S, streptomycin; TET, tetracycline. No significant association was detected between antibiotic resistance and type of animal (sick or healthy) (*p* > 0.05).

Forty-seven ESBL-producing *E. coli* isolates were detected among the 51 CTX^R^ isolates, recovered from 32 sick and 15 healthy dogs (frequencies of 13.7% and 11.8%, respectively). The phenotypes of antibiotic resistance for these ESBL-producing isolates are shown in Table 1 and the rates of antibiotic resistance of these isolates depending on their origin (sick or healthy dogs) are represented in Figure 1. No statistical difference could be established between the origin of the strain (healthy or sick dog) and the resistance to different antibiotics (*p* > 0.05) (Figure 1).

**Figure 2:**
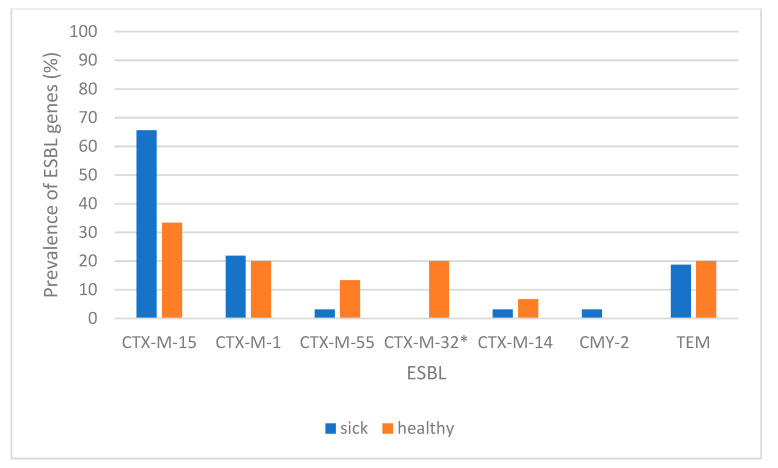
Distribution of ESBL-encoding genes from *E. coli* isolates in sick and healthy dogs. Gene encoding β-lactamases with *p* < 0.05 is indicated with (*).

Different variants of *bla*_CTX-M_ genes were detected among 45 of these 47 ESBL-producing isolates (95.4%): *bla*_CTX-M-15_ (*n* = 26 isolates), *bla*_CTX-M-1_ (*n* = 10), *bla*_CTX-M-32_ (*n* = 3), *bla*_CTX-M-55_ (*n* = 3), *bla*_CTX-M__-14_ (*n* = 2), and *bla*_CTX-M_ (*n* = 1, no variant determined) (Table 1). Figure 2 shows the distribution of the ESBL variants depending on the origin of the isolates; no statistical difference could be established between the origin of the strain (healthy or sick dog) and the ESBL type (*p* > 0.05) (Figure 2), except for CTX-M-32, in which this relation was present (it was detected just in healthy dogs).

The two remaining ESBL-positive isolates were revealed negative to all ESBL genes under study. Furthermore, a *bla*_TEM_ gene was detected in eight *bla*_CTX-M_-producing isolates. On the other hand, six ESBL-positive isolates showed cefoxitin-resistance (FOX^R^), and the *bla*_CMY-2_ gene was detected in one CTX-M-15-producing isolate obtained from a sick dog; the others ESBL-positive-FOX^R^ isolates were negative for *bla*_CMY-2_ and *bla*_DHA_ genes by PCR. Among the ESBL-positive isolates, resistance to tetracycline was mediated by the *tetA* (24 isolates) and/or *tet*B genes (Table 1). 

Two of the four CTX^R^ and ESBL-negative isolates were CMY-2-producers (qAmpC type), and they were recovered from a healthy and a sick dog (one each) (Table 2). We could not detect the mechanisms of CTX^R^ in the two remaining ESBL-negative isolates. None of the CTX^R^ *E. coli* isolates carried the *mcr*-1 gene (related to colistin resistance). 

Moreover, other β-lactamases genes such as *bla*_VEB_*, bla*_NDM,_ *bla*_OXA-48,_ and *bla*_VIM_ were tested by PCR/sequencing but all isolates were revealed to be negative. Furthermore, the *stx*_1,2_ genes related to Shiga toxin-producing *E. coli* (STEC) were not detected among our isolates.

The ESBL-positive isolates were ascribed to phylogenetic groups B_1_ (*n* = 21 isolates), A (*n* = 14), D (*n* = 7), and B_2_ (*n* = 5, two of them CTX-M-15-producers, typed as ST131) (Table 1). Furthermore, the four ESBL-negative isolates belonged to phylogroups D (*n* = 3, including the two CMY-2 producers) and A (*n* = 1) (Table 2).

MLST analysis, which was performed in thirteen representative *E. coli* isolates (based on the antimicrobial-resistance phenotype), revealed ten different lineages (ST/phylogenetic-group/β-lactamase): ST131/B2/CTX-M-15 (*n* = 2, from sick dogs, one from Lisbon and another from Bragança hospitals), ST617/A/CTX-M-55 (*n* = 1, from a healthy dog), ST3078/B_1_/CTX-M-32 (*n* = 1, from a healthy dog from the kennel), ST57/D/CTX-M-1 (*n* = 1, from a sick dog from Vouga clinic), ST12/B_2_/CTX-M-15 (*n* = 2 sick dogs, one from Vila Real and another from Lisbon), ST6448/B_1_/CTX-M-15 (*n* = 2 sick dogs, one of them CMY-2 positive and both from Lisbon), ST542/A/CTX-M-14 (*n* = 1, from a healthy dog), ST5766/A/CTX-M-32 (*n* = 1, from a healthy dog), and ST115/D/CMY-2 (*n* = 1, from a sick dog from Vouga clinic); moreover, one CMY-2-producing *E. coli* isolate of phylogroup D obtained in a healthy dog, presented a new combination of alleles (*fum*C (26), *adk* (4), *pur*A (5), *icd* (25), *gyr*B (2), *rec*A (2) and *mdh* (5)), rendering a new ST (Table 1).

## 4. Discussion

Regarding the Portuguese situation, the prevalence of ESBL-producing *E. coli* isolates in healthy dogs obtained in this work is similar to previous studies performed in dogs and cats [12,22,23] in the South and the North of Portugal. Worldwide, this prevalence was lower than the ones obtained with faecal samples of healthy dogs in Germany, Brazil, or China (24–29%) [15,37,38], but it is similar to the results of previous studies performed in Tunisia and France (12.7–17%) [11,39]. These differences could be explained by differences in the epidemiology of ESBL genes among different countries, considering the year in which the studies were performed, but we cannot discard methodological effects in the different studies. 

Five types of CTX-M ESBLs were detected, indicating a high diversity of CTX-M genes (mainly *bla*_CTX-M-15_ gene) among the CTX^R^ *E. coli* isolates; these results are in accordance with a previous study done in Portugal on healthy dogs [12]. This *bla*_CTX-M-15_ gene was also the most frequently detected in *E. coli* isolated from dogs in different countries [3,15,40]. The CTX-M-1- and CTX-M-15-encoding genes were also detected among *E. coli* canine isolates in Italy [41] and Denmark [13], which are in agreement with our data. The same variants of CTX-M genes were observed in a recent study conducted on healthy humans in Spain [42]. Moreover, during the last few years, new variants are becoming more common, in particular CTX-M-55 [3], especially from companion animals in Asian countries [43].

In the past, the *bla*_CTX-M-15_ gene was mainly associated with strains of human origin while *bla*_CTX-M-1_ was the major CTX-M sub-type among livestock and companion animal isolates in Europe [15,41]. Actually, this close correspondence is no longer so obvious, and our results confirm these data. A further study should be implemented to determine the ESBL gene in the two uncharacterized ESBL-producing isolates.

In this study, the CMY-2 gene was the qAmpC β-lactamase type found among two CTX^R^-ESBL-negative isolates and one ESBL-producing isolate, and it has been previously reported among *E. coli* strains from healthy and sick pets worldwide [20,23,39,44]. The detection of *tet*A and/or *tet*B genes in most of our tetracycline-resistant isolates seem to be similar to the results obtained by Costa et al. [45] from dogs, in Northern Portugal.

In this work, the most common phylogenetic groups among our isolates were B_1_ and A, these being the phylogroups more associated with commensal *E. coli* both in humans and in dogs, as well as in other animals [11,13]. On the other hand, isolates belonging to phylogroup B_2_ and D are more likely to be recovered from extra-intestinal infections of companion animals [4]. An interesting study related to 78 dogs that visited a veterinary hospital in Northern Portugal (either for a normal checkout or in case of disease) revealed the prevalence of *E. coli* isolates of groups A (*n* = 19), D (*n* = 9), and B_1_ (*n* = 7) [46], similar to our observation. So, the carriage of ESBL/qAmpC producing *E. coli* of these phylogroups in the gastrointestinal tract suggests a potential reservoir of MDR ESBL-producing bacteria in dogs.

Regarding the MLST results, the pandemic virulent *E. coli* ST131-B2 clone was detected among two isolates of sick dogs tested in this study. It is important to note that this clone was widely detected in pets [47,48], including in sick dogs in Portugal [17,49].

On the other hand, we detected one *E. coli* strain, ST57/D/CTX-M-1, that was recently detected in Portugal (associated with CMY-2 gene) in a dog with a UTI from a Lisbon hospital [17]. Similarly, the same lineage was identified in a faecal isolate from a healthy dog in Mexico, characterized as CMY-2/ST57/D) [50].

The frequency of the ST6448 lineage, which was observed in two sick dogs in this study, is considered an infrequent clone in humans and companion animals. This lineage was also found among a vulture faecal sample from Canary Islands [51]. To our knowledge, there is only one previous report related to the detection of this clone in humans, which was recently reported in healthy children from Sweden [52].

Additionally, our data indicate the presence of *E. coli* ST12/B2/CTX-M-15, which should be considered an agent of high clinical relevance for humans and animals. Furthermore, the ST12 lineage (associated with CMY-2) was identified in healthy dogs from Spain [6], Brazil [2], and France [11]. Furthermore, this lineage was found among isolates from children with a febrile UTI in France [53] and in healthy humans in Spain [42]. These findings highlight the dissemination of ST12 lineage and its presence in animal and human’ isolates.

To our knowledge, the ST617 lineage (clonal complex ST10) was identified for the first time in pets from Portugal in this study. CTX-M-15-producing *E. coli* isolates of sequence type ST617/phylogroup A have been reported in sick dogs in France [40] and in hospitalized patients in Tunisia [54,55]. Similarly, Rocha-Gracia et al. [50] identified the same lineage among a faecal isolate from healthy dogs in Mexico (ST617/A/CTX-M-15). According to a recent study, Gauthier, et al. [56] found this lineage in four isolates from dogs in France harbouring carbapenemase genes. Furthermore, this clone was widely disseminated.

The ST542 lineage detected in one of the healthy dogs is not commonly reported; however, this clone was found in a farmworker from Germany [57] and in a pig in Australia [58]. On the other hand, an ST115/CMY-2 isolate (found in a sick dog from the Vouga clinic) was previously reported among chickens and human patients in Germany [47].

We also detected a ST5766/A/CTX-M-32 isolate in a healthy dog; this clone is unusual, and it was previously reported in broilers’ osteomyelitis in Brazil [59]. To our knowledge, this is the first report of the ST5766 clone among pets, and the first detection in Europe. In this study, we also found an *E. coli* isolate, ST3078/B1/CTX-M-32, recovered from a healthy dog from a kennel. To our knowledge, the only unique previous study related to the ST3078 lineage was found in wastewater in Eastern France [60]. This suggests that the environment likely plays a role in the spread of ESBL-producing *E. coli* isolates in the community, associated with a One Health approach (human-animals-environment). Importantly, a new combination of alleles was found in an isolate of a healthy dog, rendering a new ST.

The use of β-lactams in the clinical practice of veterinary medicine may be considered one of the reasons for the high incidence of ESBL-producers worldwide. Thus, pets can be a significant source of ESBL/qAmpC-producing *E. coli* isolates. Considering the prevalence of ESBLs (notably the large reservoir in dogs of *E. coli* isolates with genes encoding CTX-M-15 and CTX-M-1, or CMY-2 β-lactamases), there is a serious and plausible risk of future acquisition of these resistant genes by their owners.

## 5. Conclusions

Antimicrobial resistance can make infections difficult to treat, which represents a global public health problem, due to the negative consequences for human health. This study shows that healthy and sick dogs are frequent carriers of faecal ESBL-producing *E. coli* strains, harbouring different variants of *bla*_CTX-M_ genes (mostly *bla*_CTX-M-15_ and *bla*_CTX-M-1_), and presenting a high genetic MLST diversity (including the ST131/B2 lineage). Although at a lower rate, the *bla*_CMY-2_ gene was also found. This fact suggests the implication of mobile genetic elements in the dissemination of this relevant mechanism of resistance. This underlies the complexity of the antimicrobial resistance of bacteria occurring in dogs and the possible interspecies transmission between humans, domestic animals, and into the environment, important knowledge given the One-Health approach.

## Figures and Tables

**Table 1:** Phenotypic and molecular features of the 47 ESBL-producing *E. coli* isolates recovered from healthy and sick dogs in Portugal.

Isolate Number	Origin ^a^	Sick/Healthy	Gender ^b^	Age ^c^	Breed ^d^	Phenotype of Antibiotic Resistance ^e^	β-Lactamases	Other Genes and Integrons ^f^	PG ^g^	MLST ^h^
X605	HV Lisboa	Sick	M	15A	UD	AMP, CTX, ATM, CHL, CIP, TET	CTX-M-15	*tet*(A)	B1	ST6448
X614	HV Lisboa	Sick	F	2A	UD	AMP, AUG, FOX, CTX, CAZ, ATM, CHL, CIP, TET	CTX-M-15, CMY-2	*tet*(A)	B1	ST6448
X607	HV Lisboa	Sick	F	1A	UD	AMP, AUG, CTX, CAZ, ATM, CIP, TOB, CN, S, TET	CTX-M-15	*tet*(A)	B2	ST131
X610	HV Lisboa	Sick	F	1,5A	UD	AMP, CTX, CAZ, ATM, CIP, S, TET	CTX-M-15	*tet*(A)	B2	ST12
X603	CV Bragança	Sick	F	10A	UD	AMP, AUG, CTX, CAZ, ATM, CIP, TOB, CN, S, TET	CTX-M-15	*tet*(A)	B2	ST131
X602	CV VR	Sick	F	3A	UD	AMP, AUG, CTX, CHL, TOB, CN, S, TET	CTX-M-15	*tet*(A)	B2	ST12
X558	Kennel	Healthy	M	2A	Labrador	AMP, AUG, CTX, CAZ, CIP, SXT, S, TET	CTX-M-15, TEM	*int*1	B1	NT
X562	Kennel	Healthy	F	4M	UD	AMP, CTX, CIP, SXT, S, TET	CTX-M-15, TEM	*tet*(A), *int*1	B1	NT
X569	HVTM	Sick	M	4A	UD	AMP, AUG, CTX, CAZ, ATM, CIP, SXT, TOB, CN, S, TET	CTX-M-15, TEM	*tet*(A), *tet*(B), *int*1	A	NT
X575	CV Transm	Sick	M	4A	UD	AMP, AUG, CTX, CAZ, ATM, CIP, SXT, TOB, CN, TET	CTX-M-15, TEM	*tet*(A), *tet*(B), *int*1	A	NT
C10151	HV Lisboa	Sick	F	11A	UD	AMP, AUG, CTX, TET	CTX-M-15, TEM	ND	A	NT
X550	HD	Healthy	F	8A	UD	AMP, AUG, CTX, CAZ, ATM, CHL, CIP, SXT, TET	CTX-M-15	*tet*(A), *int*1	B1	NT
X556	HD	Healthy	F	14A	Yorkshire	AMP, AUG, FOX, CTX, ATM	CTX-M-15	ND	B1	NT
X563	Kennel	Healthy	M	5A	Rottweiler	AMP, AUG, CTX, TET	CTX-M-15	ND	A	NT
X588	HVTM	Sick	M	5A	UD	AMP, AUG, CTX, CAZ, ATM, CHL, CN, TET	CTX-M-15	*tet*(A)	D	NT
X598	HVTM	Sick	M	6A	Russell Terrier	AMP, AUG, CTX, CAZ, ATM, CHL, CIP, SXT, TET	CTX-M-15	ND	B1	NT
X576	HV Lisboa	Sick	M	15A	UD	AMP, CTX, CAZ, ATM, CHL, CIP, SXT, TET	CTX-M-15	*tet*(A), *int*1	D	NT
X577	HV Lisboa	Sick	F	6M	UD	AMP, AUG, CTX, CAZ, ATM, CHL, CIP, SXT, TET	CTX-M-15	*tet*(A), *int*1	B1	NT
X578	HV Lisboa	Sick	M	13A	UD	AMP, AUG, FOX, CTX, CAZ, ATM, CHL, CIP, SXT, TOB, TET	CTX-M-15	*tet*(A)	B1	NT
X580	HV Lisboa	Sick	F	5A	UD	AMP, CTX, CAZ, ATM, CHL, CIP, SXT, TET	CTX-M-15	*int*1	B1	NT
X584	HV Lisboa	Sick	M	5A	UD	AMP, CTX, CAZ, ATM, CHL, CIP, SXT, TET	CTX-M-15	*tet*(A), *int*1	B1	NT
X604	HV Lisboa	Sick	M	12A	UD	AMP, AUG, CTX, ATM	CTX-M-15	ND	D	NT
X618	HV Lisboa	Sick	F	2A	UD	AMP, AUG, FOX, CTX, CAZ, ATM, CHL, CIP, SXT, TET	CTX-M-15	*tet*(A), *int*1	B1	NT
X620	HV Lisboa	Sick	M	9A	UD	AMP, AUG, CTX, CAZ, ATM, CIP, SXT, TOB, CN, S, TET	CTX-M-15	*tet*(A), *int*1	D	NT
X622	HV Lisboa	Sick	M	3A	UD	AMP, AUG, FOX, CTX, CAZ, ATM, CIP, SXT, S, TET	CTX-M-15	ND	A	NT
X599	CV Bragança	Sick	M	7A	Rodengo	AMP, AUG, FOX, CTX, CAZ, ATM, CHL, CIP, TET	CTX-M-15	ND	B1	NT
C10264	CV Vouga	Sick	F	9M	Pincher	AMP, CTX, CAZ	CTX-M-1	ND	D	ST57
X554	HVTM	Sick	M	1A	Labrador	AMP, CTX, CAZ, TET	CTX-M-1, TEM	ND	A	NT
X557	Kennel	Healthy	F	3A	Serra Estrela	AMP, AUG, CTX, CAZ, TOB, AK, S	CTX-M-1	ND	B1	NT
X559	Kennel	Healthy	F	3M	UD	AMP, AUG, CTX, CAZ	CTX-M-1	ND	D	NT
X560	Kennel	Healthy	M	1A	Labrador	AMP, AUG, CTX, CAZ, TET	CTX-M-1	ND	B1	NT
X581	HV Lisboa	Sick	M	14A	UD	AMP, AUG, CTX, CAZ, TET	CTX-M-1	*tet*(A)	B1	NT
X611	HV Lisboa	Sick	M	5A	UD	AMP, CTX, CAZ, ATM, CHL, CIP, CN, TET	CTX-M-1	*tet*(A)	D	NT
X616	HV Lisboa	Sick	M	3M	UD	AMP, AUG, CTX, CAZ, TET	CTX-M-1	ND	B1	NT
X617	HV Lisboa	Sick	F	3A	UD	AMP, CTX, CAZ, TET	CTX-M-1	*tet*(A)	B1	NT
C10265	CV Bragança	Sick	M	1A	UD	AMP, CTX, CAZ, S	CTX-M-1	ND	A	NT
X555	HD	Healthy	M	6A	Pastor alemão	AMP, CTX, CAZ, ATM, CIP, SXT, TOB, CN, TET	CTX-M-55	*tet*(B), *int*1	A	ST617
X568	HD	Healthy	M	7A	UD	AMP, AUG, CTX, CAZ, ATM, CHL, CIP, SXT, TET	CTX-M-55	*tet*(A), *int*1	B1	NT
C10149	HVTM	Sick	F	1,5A	UD	AMP, CTX, CAZ, CHL, TOB, CN, S, TET	CTX-M-55, TEM	*tet*(A)	A	NT
X573	HD	Healthy	M	1A	UD	AMP, AUG, CTX, CAZ, ATM, CHL, SXT, S, TET	CTX-M-32	*int*1	A	ST5766
X561	Kennel	Healthy	M	2A	Gado transm.	AMP, AUG, CTX, ATM, CHL, CIP, SXT, TOB, CN, S, TET	CTX-M-32	*tet*(A), *int*1	B1	ST3078
X571	HD	Healthy	M	1A	UD	AMP, AUG, CTX, CAZ, CHL, SXT, S, TET	CTX-M-32, TEM	*tet*(B), *int*1	B1	NT
X572	HD	Healthy	F	1A	UD	AMP, AUG, CTX, CAZ, S, TET	CTX-M-14	*tet*(B)	A	ST542
X574	CVTransm	Sick	M	4A	UD	AMP, CTX, CHL, SXT, TOB, CN, S, TET	CTX-M-14	ND	A	NT
X565	HD	Healthy	M	6A	UD	AMP, CTX, ATM, CIP, SXT, TOB, CN, S, TET	CTX-M-variant	ND	A	NT
C10147	HVLisboa	Sick	M	7A	UD	AMP, CTX, CHL, SXT, CN, S, TET	TEM-1	*tet*(A), *int*1	B2	NT
X587	HVTM	Sick	M	2A	Bulldog Francês	AMP, CTX, ATM, CHL, SXT, CN, S, TET	No *bla* genes	*int*1	A	NT

^a^ HD- healthy dogs from their owners; HVTM- *Hospital Veterinário de Trás os Montes* (Vila Real); Kennel-healthy dogs from the kennel (Vila Real); CV Transm- Clínica Veterinária Transmonvete (Vila Real, Portugal); HV Lisboa- *Hospital Veterinário de São Bento (Lisboa)*; CV Vouga- Clínica Veterinária do Vouga (Sever do Vouga, Portugal); CV Bragança- Clínica Veterinária de Macedo de Cavaleiros (Bragança, Portugal); CV VR- Clínica Veterinária dos Quinchosos (Vila Real, Portugal); ^b^ female; M-male; ^c^ A- years; M- months; ^d^ UD- undetermined dog breed; ^e^ AMP, ampicillin; AUG, amoxicillin–clavulanic acid; FOX, cefoxitin; CTX, cefotaxime; CAZ, ceftazidime; ATM, aztreonam; CHL, chloramphenicol; CIP, ciprofloxacin; TOB, tobramycin; AK, amikacin; CN, gentamicin; SXT, trimethoprim–sulfamethoxazole; S, streptomycin; TET, tetracycline; ^f^ ND: not detected; ^g^ Phylogroups; ^h^ MLST-Multilocus Sequence Typing; NT: not tested.

**Table 2:** Phenotypic and molecular features of ESBL-negative *E. coli* isolates recovered from healthy and sick dogs in Portugal.

Isolate Number	Origin ^a^	Gender ^b^	Age ^c^	Breed ^d^	Antimicrobial Resistance Phenotype ^e^	Resistance Genotype	Other Resistance Genes ^f^	PG ^g^	MLST ^h^
X551	HD	F	24M	Golden Retriever	AMP, CTX	CMY-2	ND	D	New ST *
X567	CV Vouga	F	8A	UD	AMP, AUG, FOX, CTX, CAZ, CIP, S, TET	CMY-2, TEM	*tet*(A)	D	ST115
X549	HVTM	F	6A	Leão Rodesea	AMP, AUG, CTX	ND	ND	D	NT
C10266	HV Lisboa	F	6A	UD	AMP, AUG, FOX, CTX, CAZ, ATM, NA, CIP, SXT, S, TET	ND	*tet*(B)	A	NT

^a^ HD- healthy dogs from their owners; HVTM- *Hospital Veterinário de Trás os Montes* (Vila Real); Kennel- healthy dogs from kennel (Vila Real); CV Transm- Clínica Veterinária Transmonvete (Vila Real, Portugal); HV Lisboa- *Hospital Veterinário de São Bento (Lisboa)*; CV Vouga- Clínica Veterinária do Vouga (Sever do Vouga, Portugal); CV Bragança- Clínica Veterinária de Macedo de Cavaleiros (Bragança, Portugal); CV VR- Clínica Veterinária dos Quinchosos (Vila Real, Portugal); ^b^ F-female; M-male; ^c^ A- years; M- months; ^d^ UD- undetermined dog breed; ^e^ AMP, ampicillin; AUG, amoxicillin–clavulanic acid; FOX, cefoxitin; CTX, cefotaxime; CAZ, ceftazidime; ATM, aztreonam; NA, nalidixic acid; CIP, ciprofloxacin; SXT, trimethoprim–sulfamethoxazole; S, streptomycin; TET, tetracycline; IMP, imipenem; ETP, ertapenem. ^f^ ND: not detected; ^g^ Phylogroups; ^h^ MLST-Multilocus Sequence Typing; NT: not tested. * New ST allelic combination: *fum*C (26), *adk* (4), *pur*A (5), *icd* (25), *gyr*B (2), *rec*A (2), and *mdh* (5).

## Data Availability

The data presented in this study are available in Supplementary Material.

## Supplementary Materials

The following are available online at https://www.mdpi.com/article/10.3390/antibiotics10081013/s1, Figure S1: Geographic location of the different areas where the faecal samples from sick dogs were collected in Portugal. Table S1: Primers sequences and PCR conditions used for genes encoding antibiotic resistance in *E. coli*.
