# Supplementary material for: Antimicrobial Resistance Genes and Diversity of Clones among Faecal ESBL-Producing Escherichia coli Isolated from Healthy and Sick Dogs Living in Portugal
Source: Antibiotics (Basel). 2021 Aug 20;10(8):1013. doi: 10.3390/antibiotics10081013 (PMC8388948; doi:10.3390/antibiotics10081013)
Supplement: Supplementary file 1 [file antibiotics-10-01013-s001.zip › antibiotics-1294598-supplementary.pdf]

Supplementary Material

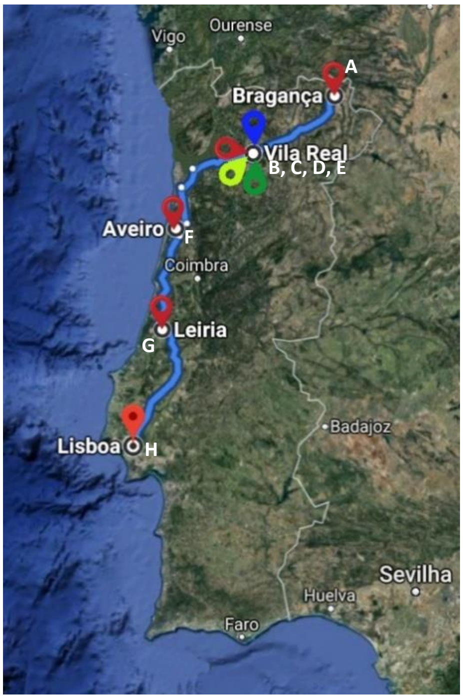

Figure S1. – Geographic location of the different areas where the faecal samples from sick dogs were collected in Portugal. A – *Clínica Veterinária de Macedo de Cavaleiros (Bragança);* **B –** Kennel (Vila Real); **C -** *Clínica Veterinária dos Quinchosos (Vila Real);* ***D*** *– Transmonvete (Vila Real*), **E –** *Hospital Veterinário de Trás os Montes HVTM* (Vila Real); ***F*** *- Clínica Veterinária do Vouga (Aveiro)*; **G -** *Clínica Veterinária da Guia (Leiria);* **H** *– Hospital Veterinário de São Bento (Lisboa).*

Table S1. – Primers sequences and PCR conditions used for genes encoding antibiotic resistance in *E. coli*.

Gene	Antibiotics	Primers sequences (5')→(3')	PCR conditions	Size band (bp)	References
bla $_{TEM}$	***β-lactams***	ATTCTTGAAGACGAAAGGGC<br>ACGCTCAGTGGAACGAAAAC	94ºC 3 min.<br>94ºC 1 min.<br>60ºC 1 min. (30 cycles)<br>72ºC 1 min.<br>72ºC 10 min.	1150	(Pitout *et al.*, 1998)
bla $_{SHV}$	***β-lactams***	CACTCAAGGATGTATTGTG<br>TTAGCGTTGCCAGTGCTCG	96ºC 5 min.<br>96ºC 15 s<br>52ºC 15 s (24 cycles)<br>72ºC 2 min.<br>72ºC 5 min.	885	(Pitout *et al.*, 1998)
bla $_{CTX-M-9}$<br>***(Group 9)***	***β-lactams***	GTGACAAAGAGAGTGCAACGG<br>ATGATTCTCGCCGCTGAAGCC	94ºC 3 min.<br>94ºC 45 s<br>62ºC 45s (35 cycles)<br>72ºC 45 s<br>72ºC 10 min.	857	(Zong *et al.*, 2008)

bla**$_{CTX-M-1}$* ***(Group 1)	***β-lactams***	GTTACAATGTGTGAGAAGCAG<br>CCGTTTCCGCTATTACAAAC	94ºC 7 min.<br>94ºC 50 s.<br>50ºC 40 s. (35 cycles)<br>68ºC 1 min.<br>68ºC 5 min.	1017	(Zong *et al.*, 2008)
***bla**$_{CTX-M-UNIVERSAL}$*	***β-lactams***	CGATGTGCAGTACCAGTAA<br>TTAGTGACCAGAATCAGCGG	94ºC 5 min.<br>94ºC 30 s<br>60ºC 30 s (35 cycles)<br>72ºC 1 min.<br>72ºC 5 min.	585	(Zong *et al.*, 2008)
***bla**$_{CMY-2}$*	***β-lactams***	GATTCCTTGGACTCTTCAG<br>TAAAACCAGGTTCCCAGATAGC	95ºC 3 min.<br>95ºC 30 s<br>53ºC 30 s (35 cycles)<br>72ºC 30 s<br>72ºC 3 min.	1807	(Hassen *et al.*, 2019)
***bla**$_{KPC2/3}$*	***carbapenems***	GTATCGCCGTCTAGTTCTGC<br>GGTCGTGTTTCCCTTTAGCC	94ºC 5 min.<br>94ºC 30 s<br>58ºC 30 s (25 cycles)<br>72ºC 1 min.<br>72ºC 7 min.	638	(Ellington *et al.*, 2007)
***bla**$_{OXA-48}$*	***carbapenems***	TTGGTGGCATCGATTATCGG<br>GAGCACTTCTTTTGTGATGGC	96ºC 5 min.<br>96ºC 1 min.<br>61ºC 1 min. (35 cycles)<br>72ºC 2 min.	743	(Ruiz, 2011)

			72ºC 10 min.		
***bla*$_{VEB}$**	***carbapenems***	CGACTTCCATTTCCCGATGC<br>GGACTCTGCAACAAATACGC	94ºC 5 min.<br>94ºC 30 s<br>58ºC 30 s (25 cycles)<br>72ºC 1 min.<br>72ºC 7 min.	643	(Ellington *et al*., 2007)
***bla*$_{VIM}$**	***carbapenems***	GATGGTGTTTGGTCGCATA<br>CGAATGCGCAGCACCAG	94ºC 5 min.<br>94ºC 30 s.<br>52ºC 40 s (36 cycles)<br>72ºC 50 s.<br>72ºC 5 min.	390	(Ellington *et al*., 2007)
***bla*$_{NDM}$**	***carbapenems***	AATATTATGCACCCGGTCGCG<br>CATCACGATCATGCTGGCCTT	94ºC 5 min.<br>94ºC 1 min.<br>52ºC 1 min (30 cycles)<br>72ºC 1 min.<br>72ºC 7 min.	804	(Ellington *et al*., 2007)
tetA	***tetracycline***	GTAATTCTGAGCACTGTCGC<br>CTGCCTGGACAACATTGCTT	95ºC 5 min.<br>95ºC 30 s<br>62ºC 30 s (23 cycles)<br>72ºC 45 s<br>72ºC 7 min.	937	(Ellington *et al*., 2007)

tetB	***tetracycline***	CTCAGTATTCCAAGCCTTTG<br>CTAAGCACTTGTCTCCTGTT	95ºC 5 min.<br>95ºC 30 s<br>57ºC 30 s (25 cycles)<br>72ºC 20 s<br>72ºC 7 min.	416	(Ellington *et al.*, 2007)
mcr-1	***colistin***	CTTGGTCGGTCTGTAGGG<br>CGGTCAGTCCGTTTGTTC	94ºC 15 min.<br>945ºC 30 min.<br>58ºC 1,5 min. (25 cycles)<br>72ºC 1 min.<br>72ºC 10 min.	309	(Liu *et al.*, 2016)
int1	integron	GGGTCAAGGATCTGGATTTCG<br>ACATGCGTGTAAATCATCGTCG	94ºC 5 min.<br>94ºC 30 s<br>62ºC 30 s (30 cycles)<br>72ºC 1 min.<br>72ºC 8 min.	483	(Vinué, 2008)
